# A Framework for Reconstructing Archaeological Networks Using Exponential Random Graph Models

**DOI:** 10.1007/s10816-019-09423-z

**Published:** 2019-08-19

**Authors:** Viviana Amati, Angus Mol, Termeh Shafie, Corinne Hofman, Ulrik Brandes

**Affiliations:** 1 Department of Humanities, Social and Political Sciences, Social Networks Lab, ETH Zurich, Weinbergstrasse 109, 8092 Zurich, Switzerland; 2 Leiden University Centre for Digital Humanities, Nonnensteeg 1-3, 2311 VJ Leiden, The Netherlands; 3 Department of Social Statistics, Mitchell Centre for Social Network Analysis, University of Manchester, Humanities Bridgeford Street, Manchester, M139PL UK; 4 Faculty of Archaeology, Leiden University, Einsteinweg 2, 2333 CC Leiden, The Netherlands

**Keywords:** Early Ceramic Age, Caribbean networks, Exponential random graph models, Network reconstruction

## Abstract

Reconstructing ties between archaeological contexts may contribute to explain and describe a variety of past social phenomena. Several models have been formulated to infer the structure of such archaeological networks. The applicability of these models in diverse archaeological contexts is limited by the restricted set of assumptions that fully determine the mathematical formulation of the models and are often articulated on a dyadic basis. Here, we present a general framework in which we combine exponential random graph models with archaeological substantiations of mechanisms that may be responsible for network formation. This framework may be applied to infer the structure of ancient networks in a large variety of archaeological settings. We use data collected over a set of sites in the Caribbean during the period AD 100–400 to illustrate the steps to obtain a network reconstruction.

**Electronic supplementary material:**

The online version of this article (10.1007/s10816-019-09423-z) contains supplementary material, which is available to authorized users.

## Introduction

Empirical and theoretical studies of networks based on archaeological data are on a rapid rise (Brughmans 2013; Collar et al. 2015; Knappett 2011). However, relatively few studies attempt to infer the structure of past networks. Ties between archaeological contexts (*e.g.* sites) or attributes of these contexts (*e.g.* site assemblages and artefacts) may contribute to the analysis of the structural characteristics of networks in the past and the evaluation of their impact on a variety of past social phenomena. Examples include the diffusion of innovations (Mol 2013, 2014; Roux and Manzo 2018), direct contact (Boomert 2000; Hofman et al. 2014), exchange of goods (Hofman et al. 2007; Knippenberg 2007), central place redistribution (Crock 2000), the mobility of groups of people or individuals (Laffoon et al. 2017; Rouse 1992) and their interrelations—*e.g.* human mobility and the exchange of goods and ideas (Hofman et al. 2010).

Two main approaches have been adopted to infer the structure of past networks (Östborn and Gerding 2014). One relies on the assumption that similarity in site assemblages is a proxy for the existence of ties (Coward 2010, 2013; Mills et al. 2013; Mol 2014; Habiba et al. 2018) so that “the broken links of a ruined network [are inferred] from observable distributions and patterns of association in the archaeological record” (Sindbaek 2013, p. 71). The other one focusses on the processes that might have created ties in the past and consists in specifying a model from which plausible networks are generated. The formulation of this model hinges on the assumptions archaeologists have about the formation of the relation(s) of interest (Bevan and Wilson 2013; Knappett et al. 2008; Terrell 1977). Here, we focus on the second approach and, in particular, on tie-based models, the definition of which depends on assumptions articulated at the tie level, as opposed to agent-based models, which are formulated based on propositions articulated at the node level (Graham 2006; Wurzer et al. 2015).

Several tie-based models have been used for reconstructing archaeological networks, among them maximum distance networks (Evans et al. 2012), proximal point analysis (Broodbank 2002; Terrell 1977), gravity models (Conolly and Lake 2006; Hodder 1974; Johnson 1977) and ariadne (Rivers et al. 2011). The applicability of these models in diverse archaeological contexts is limited by their mathematical formulation which is fully determined by the propositions those models entail. Different sets of tie formation assumptions require the formulation of new generative models.

Maximum distance networks, proximal point analysis, gravity models and ariadne also postulate that the existence of a tie between any two archaeological contexts *i* and *j* depends on entity attributes measured at the node level (*e.g.* size) or at the dyadic level (*e.g.* geographical location), as well as the constraints imposed on them. Therefore, they assume that the mechanisms that might have generated a network act only on a dyadic base. The assumption of tie independence overlooks more complex processes of network formation suggesting that networks are the outcomes of interdependent interactions embedded in a certain environment—rather than outcomes of interactions taking place in a vacuum of dyadic relations.

Archaeological propositions concerning the formation of ties among diverse archaeological entities (*e.g.* sites, households, cities or regions) that, more or less explicitly, embody the idea of tie dependence cannot be represented using the models mentioned above. Examples of archaeological propositions implying tie dependence relate to transitivity, and its opposite, indirect exchange in trade networks. Given three archaeological contexts *i*, *j* and *k*, transitivity implies that if *i* exchanges goods with *j* and *j* with *h*, it is likely that *i* will start exchanging goods with *h* as well. Contrary to transitivity, indirect exchange indicates that it is less likely that *i* will start exchanging goods with *h* as well (see, *e.g.* Blake (2014), for more details and discussion).

In this paper, we propose to use standard statistical models for the analysis of networks to reconstruct ancient networks. In particular, we consider exponential random graph models (ERGMs) (Lusher et al. 2012; Robins et al. 2007; Wasserman and Pattison 1996). These models have already been used to reconstruct structurally efficient networks of contacts, *i.e.* plausible network scenarios where contacts between archaeological contexts were regulated by broker sites (Amati et al. 2018). Building on this previous work, we demonstrate that the applicability of ERGMs is not limited to the reconstruction of structurally efficient networks; rather, ERGMs constitute a flexible family of models that allows researchers to reconstruct networks given a variety of assumptions, contexts and relations. The derivation of the mathematical formulation of ERGMs does not depend on the context and the particular assumptions about the mechanisms regulating the formation of ties in the past. Moreover, ERGMs subsume maximum distance networks, proximal point analysis and gravity models as special cases and therefore have the potential of providing a unified model for inferring ties among entities in the past.

In this paper, we present a general framework in which we combine ERGMs with archaeological theories of mechanisms that may be responsible for network formation. Given a set of archaeological contexts, a particular relation (*e.g.* contact or exchange) and a set of propositions describing how ties formed, the described framework permits researchers to generate plausible network scenarios. To illustrate the steps of the procedure, we used data collected over a group of sites in the Caribbean during the period AD 100–400. This period falls under the (Middle) Early Ceramic Age, but should rather be seen as a new phase in which previous ways of life (more prevalent in the so-called Archaic period) were fully transformed into what is referred to as the Ceramic Age. To reflect this dynamic, as well as to somewhat neutralize the awkward term “Archaic”, we will here refer to the specific AD100–400 period as the Archaic-Early Ceramic Interface (AECI) period.

The remainder of the paper is organised as follows. In the “Exponential Random Graph Models” section, ERGMs are introduced; the steps necessary to reconstruct archaeological networks are presented in the “ERGMs for Network Reconstruction” section. Illustrative examples of the proposed framework are provided in the “Example: an Application to the Pre-colonial Caribbean” section. The framework and its application are further discussed in the “Conclusion” section.

## Exponential Random Graph Models

### Definition

Let N  = {1, …, *n*} be a set of archaeological contexts, hereafter referred to as sites, and *R*: N  × N  → [0,1] be a binary relation between them. We represent a network as an adjacency matrix *x*, whose cell *x*_*ij*_ takes value 1 if there is a relationship between sites *i* and *j*, and 0 otherwise. When *x*_*ij*_ = 1, we say that there is a tie between *i* and *j*, or that *i* is tied to *j*. Ties are undirected (*e.g.* contact or connectedness) when the existence of a tie from *i* to *j* implies the existence of a tie from *j* to *i* (*i.e. x*_*ij*_ = 1 implies *x*_*ji*_ *=* 1). Ties are directed (*e.g.* exchange) when the existence of a tie from *i* to *j* does not imply the existence of a tie from *j* to *i* (*i.e. x*_*ij*_ = 1 does not imply *x*_*ji*_ = 1). Let *v* be the case by variable matrix containing the attributes of the archaeological contexts (*e.g.* size and location) and *w* be an array containing dyadic information (*e.g.* distance between contexts).

Exponential random graph models (ERGMs) are a class of statistical models for networks. Their mathematical formulation was originally derived by Frank and Strauss (1986) using concepts from spatial statistics, and subsequently extended by Wasserman and Pattison (1996). Later derivations based on notions of other disciplines have been proposed. In theoretical physics, and more specifically statistical mechanics, scholars have linked the behaviour and the arrangements of particles in a system to those of entities in a network and derived ERGMs from the Gibbs-Boltzmann distribution (Newman 2018a). Related to this derivation, by employing information theory, researchers have deduced the formulation of ERGMs using the maximum entropy principle (Shannon 1948; Jaynes 1957a, 1957b). More recently, Butts (2009) and Mele (2017) derived the formulation of some specifications of ERGMs from principles of game theory. For a more detailed discussion about the derivation of ERGMs, the sources cited above are recommended.

ERGMs implicitly assume that the global structure of an observed network emerges from dynamic processes acting simultaneously and representing the rules describing the formation of ties in a network. Hereafter, we refer to these dynamic processes as generative mechanisms. Examples of generative mechanisms include transitivity and homophily, suggesting that ties are formed between entities that are both tied to one or more common entities and between entities that have similar characteristics, respectively. Traces of these mechanisms in an observed network are particular patterns of ties referred to as local configurations. Table 1 displays the local configurations corresponding to the mechanisms mentioned above. Intuitively, a large number of triangles and homophilic dyads in the observed network indicates that the structure of the observed network emerges from transitivity and homophily mechanisms.

**Table 1:**
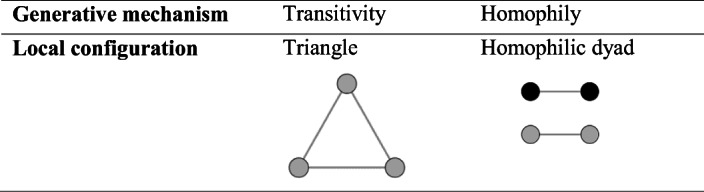
Example of the correspondence between generative mechanisms and dynamic processes for transitivity and homophily. The colours of the nodes represents different values taken by an entity attribute

Formally, ERGMs are a family of probability distributions over the space *x* of all the possible networks defined on the set of nodes N and have the form

1 $$ P\left(X=x\right)=\frac{1}{\kappa}\exp \left(\sum \limits_k{\theta}_k{s}_k\left(x,v,w\right)\right), $$

where *X* is the network random variable, *x* denotes the value taken by *X*, *θ*_*k*_ represents a parameter, *s*_*k*_(*x*,*v*,*w*) is a statistic and $\kappa ={\sum}_{x\hbox{'}\in \mathcal{X}}\exp \left(\sum \limits_k{\theta}_k{s}_k\left(x\hbox{'},v,w\right)\right)$ is a normalising constant.

The linear combination $\sum \limits_k{\theta}_k{s}_k\left(x,v,w\right)$ in Eq. (1) is the mathematical representation of the assumption that the structure of an observed network is the outcome of dynamic processes acting simultaneously. More specifically, the statistic *s*_*k*_(*x*,*v*,*w*) counts the number of local configurations of type *k* which are the traces of the *k*th generative mechanism. The sum over *k* expresses the idea that more than one mechanism may have generated the network *x*: Ties may occur due to the presence/absence of other ties, as well as the attributes of the entities or pairs of entities. This fact is mathematically represented by the arguments of the statistic *s*_*k*_, *i.e.* the network *x*, the entity attribute *v* and the dyadic information *w*. We provide a list of the most common statistics and their connection to the mechanisms they entail in the next section.

The parameter *θ*_*k*_ measures the importance of the local configurations of type *k* in determining the global structure of the network, in other words, the relative importance of a mechanism to the formation of ties. A positive (negative) value of the parameter *θ*_*k*_ indicates that a tie is more (less) likely to occur when its presence increases (decreases) the value of the statistic *s*_*k*_(*x*,*v*,*w*), thereby providing evidence for (against) the corresponding mechanism. This interpretation stems from the fact that ERGMs can be regarded as log-linear models for the binary tie random variables *X*_*ij*_ the collection of which generates the random network *X*. According to this conceptualisation, the logarithm of the ratio between the probability of a tie being present and the probability of a tie being absent, conditional on the other ties in the network, is expressed as follows:

2 $$ \log \left[\frac{P\left({X}_{ij}=1|{x}_{ij}^c;\theta \right)}{P\left({X}_{ij}=0|{x}_{ij}^c;\theta \right)}\right]=\sum \limits_k{\theta}_k\left[{s}_k\left({x}^{+ ij},v,w\right)-{s}_k\left({x}^{- ij},v,w\right)\right], $$

where *x*^*+ij*^ and *x*^*−ij*^ denote the networks with the tie *x*_*ij*_ present (*i.e. x*_*ij*_ = 1) and absent (*i.e. x*_*ij*_ = 0), respectively, whilst ${x}_{ij}^c$ represents the set of all the tie variables except *x*_*ij*_.

For those familiar with logistic regression models, Eq. (2) indicates that the parameters of an ERGM can be interpreted in a similar way to the parameters of a logistic regression model (Shennan 1997; Agresti and Kateri 2011). If *θ*_*k*_ is positive and if the presence of a tie between two nodes *i* and *j* leads to an increase in the value of the statistic *s*_*k*_(*x*,*v*,*w*), then the tie *X*_*ij*_ is more likely to be present than absent, whilst keeping all the other statistics fixed. However, it should be noted that this interpretation has only a heuristic value since the existence or the deficiency of a tie might change the value of multiple statistics at the same time. For instance, the presence of a tie between *i* and *j* increases the number of both the edges and triangles when the ties between *i* and *h*, as well as *j* and *h* exist.

### Statistics

Many statistics have been defined to specify ERGMs (Morris et al. 2008; Wang et al. 2009). Table 2 and Table 3 show the most common local configurations along with the mechanisms of tie formation that they represent. These tables also contain references to theories that have been developed in archaeology and can be easily encoded into combinations of ties.

**Table 2:**
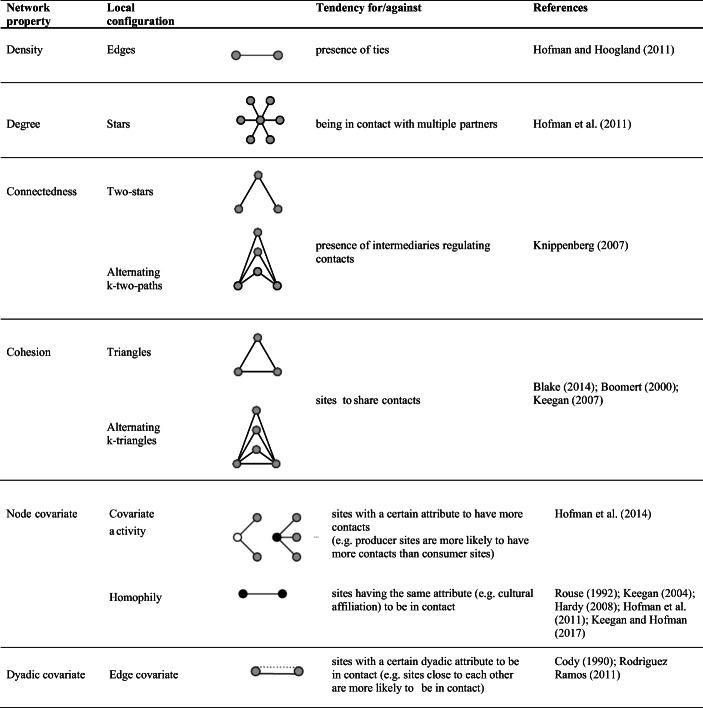
Network properties, local configurations, interpretation and references to corresponding archaeological theories for undirected relations. Node and dyadic attributes are represented by the colour of the nodes and dotted lines, respectively

**Table 3:**
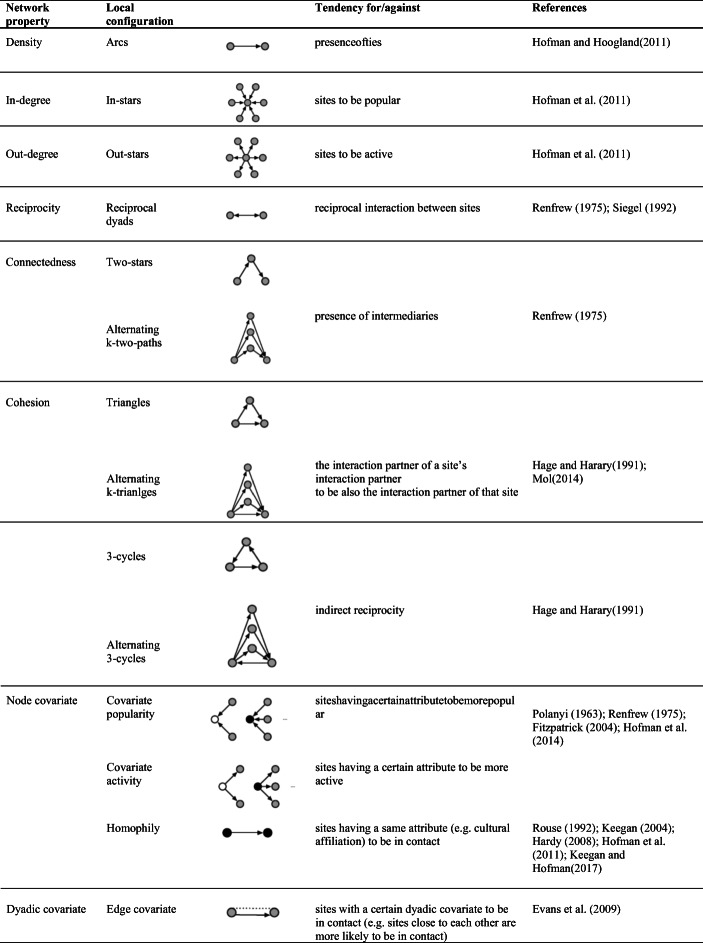
Network properties, local configurations, interpretation and references to corresponding archaeological theories for directed relations. Node and dyadic attributes are represented by the colour of the nodes and dotted lines, respectively

Statistics for directed ties are extensions of statistics for undirected ties. Therefore, in the following sections, we mainly focus on the statistics for undirected relations, and we briefly describe those for the directed case. We relate these statistics to both the network properties and the archaeological propositions they represent. We distinguish between endogenous statistics—associated with propositions concerning the existence of ties in reaction to the existence of other ties—and exogenous statistics—encoding the propositions expressing the presence of ties based on site characteristics.

#### Endogenous Statistics

Endogenous statistics model the dependence of ties on the existence of other ties. The constituent elements of a network are ties, thus the most elementary endogenous statistic counts the number of edges and pertains to the network density. The *edge* statistic describes the propensity of nodes to form ties. However, some nodes might be more prone to form ties than others. This tendency is captured by the degree distribution, with the degree being the number of ties incident to a node. The degree distribution is described by the *k-star* statistic, which counts the number of nodes connected to other *k* nodes. The number *k* ranges between 1 and *n*-1, albeit only 2-stars and 3-stars are used in practice. A statistic accounting for all the *k*-stars simultaneously is the *alternating-k-star* statistic, defined as a weighted sum of the *k*-star statistics. The *k*-star and alternating-*k*-star statistics capture the tendency of sites to be in contact with multiple partners and are measures of centralisation. For the directed case (*e.g.* exchange or flow of goods), a distinction between incoming ties and outgoing ties is operated, leading to the *in-degree* and *out-degree* statistics.

Connectedness refers to the existence of paths between any pair of nodes and assesses (direct and indirect) reachability. The *two-path* statistic is used to model this network property. It counts the number of two-paths, sequences of two ties connecting two nodes through an intermediary node. To account for multiple intermediaries between any pair of nodes, the *alternating-k-two-path* statistic is used. This statistic is a weighted sum of counts of *k*-two-paths, with *k* being the number of intermediaries between two nodes. Two-paths are used, *e.g.* to account for the “middle-man” assumption, supposing the presence of broker or intermediary sites that mediate the contacts between other sites. For the directed case, two-paths are defined as a sequence of a tie from node *i* to node *j* and a tie from node *j* to a third node *h,* with *j* being the intermediary node.

Cohesion is another important network property. It refers to the tendency of ties to cluster together. The simplest configuration representing clustering is a triangle. In many networks, triangles tend to cluster as well, forming clumps modelled by the *alternating-k-triangle* statistic. Whilst the *triangle* statistic simply counts the triangles in a network (*i.e.* the number of times two connected sites are tied to a same other node), the *alternating-k-triangle* statistic accounts for the number of neighbours shared by two connected nodes. The use of these statistics is based on the proposition that ties are formed between sites that are jointly connected to at least a third site. An example of this assumption is that sites tend to form ties within social groups, where the site’s contacts are connected to each other.

For directed relations, several types of triangles can be defined, among them *transitive triads* and *three-cycles,* which both describe the extent to which existing two-paths in a network are closed. Transitive triads refer to the tendency of nodes that are indirectly connected through a third node to directly connect. For instance, they might be used to represent the proposition that the exchange partner of a site’s exchange partner is also the exchange partner of that site. Three-cycles are an undirected form of reciprocity. The assumption that the flow from site *i* to site *j* is returned through a third site *h* translates into a three-cycle configuration. Due to this interpretation, the transitive triad and three-cycle statistics are usually used jointly to reconstruct networks characterised by hierarchy differences among nodes. Indeed, the tendency towards transitive triads and against three-cycles indicates that certain nodes have more prominent positions than others.

#### Exogenous Statistics

Exogenous statistics model the dependence of ties on monadic or dyadic site attributes.

The *covariate-activity* statistic allows to control for the dependence of the degree on node attributes. In the directed case *(e.g.* exchange), this statistic can be used to model the tendency of supplier sites to have more outgoing ties than consumer sites do.

Homophily, a mechanism referring to the similarity of connected nodes, is another classic example (McPherson et al. 2001). Two sets of statistics are available to model homophily: one counting the number of ties between nodes having the same characteristics and another counting the number of ties between pairs of nodes having different characteristics. Both statistical counts are suitable, for instance, to model the assumption that sites with the same cultural affiliation are more likely to be in contact.

Another set of statistics depends on dyadic attributes, *i.e.* the characteristics of pairs of nodes, such as geographical proximity. The corresponding statistic is a sum of ties weighted by the distance between the nodes and is used to account for the role that distance plays in regulating the existence of ties. Therefore, a widespread, because intuitive, proposition that ties between closer sites are more likely, can be modelled using this statistic.

Many other statistics can be defined as interaction effects among the statistics. An example is the interaction of two-paths with geographical distance. This interaction, as shown in one of the illustrative examples in the “Example: an Application to the Pre-colonial Caribbean” section, allows to account for the assumption that intermediary sites act on a local scale.

## ERGMs for Network Reconstruction

In this section, we describe the steps needed to reconstruct archaeological networks using ERGMs.

Given a certain relation and a set of theories concerning the mechanisms regulating the formation of ties, the first step of the procedure consists of fully specifying the model, *i.e.* choosing the statistics and the values of the corresponding parameters. The choice of the statistics requires matching the archaeological propositions to the corresponding local configurations. The previous section, jointly with Table 1 and Table 2, provides examples of this correspondence.

The values of the parameters determine how strong the tendency towards or against a specific proposition is. In general, positive (negative) values of a parameter lead to networks with high (low) values of the corresponding statistic, thereby indicating tendencies for (against) the associated proposition. However, certain combinations of parameter values (*e.g.* large positive or negative values) might lead to unrealistic network reconstructions corresponding to almost complete or empty networks. This phenomenon has been investigated in several disciplines, and it is referred to as *near-degeneracy* in statistics (Chatterjee and Diaconis 2013) and in the social science (Handcock et al. 2003; Snijders 2002; Snijders et al. 2006), and *phase transition* in physics (Newman 2003, 2018a). It follows that the choice and the calibration of the parameters are fundamental in order to avoid near-degeneracy and reconstruct networks of archaeological interest.

To avoid the pitfall of specifying a degenerate distribution concentrated only on the empty or the complete network, the following procedure was used. The parameter values of all the statics were fixed on values derived from the network literature, and then tuned so that the simulated networks have structural characteristics coherent with the archaeological evidence. A similar method was used by Amati et al. (2018), but the initial values of the parameters were obtained by estimating a fully specified ERGM on a network reconstructed using one of the previous models (*e.g.* gravity model or ariadne), and then tuning the values obtained according to the available archaeological information and the density of the resulting networks.

The second step of the procedure aims at determining a plausible network reconstruction. For a fully specified ERGM, which is assumed to be a good representation of the processes thought to have generated networks in the past, it is natural to look for the most likely network(s) as a plausible reconstruction. Thus, the second step of the procedure consists of finding the network(s) that maximises the linear combination of statistics and parameters defined by ∑_*k*_*θ*_*k*_*s*_*k*_(*x*, *v*, *w*).

Due to the large number of networks that can be defined on the set of nodes N , the maximisation of this function is difficult except in some trivial cases (*e.g.* when all the parameters are positive or negative). The solution of the maximisation problem can be approximated by using simulated annealing (Metropolis et al. 1953; Kirkpatrick et al. 1983), an algorithm that applies to the optimisation of a function on a finite set with very large size and owes its name to the annealing process in metallurgy. This algorithm avoids the trapping attraction of the local maxima of the function that needs to be maximized by scaling the function by a parameter *T*, named temperature.

Given an initial value of the temperature *T* > 0 and an initial network *x*, at each step of the algorithm, a tie *x*_*ij*_ is selected uniformly at random and a change is suggested: If the tie *x*_*ij*_ is present in the network, the proposed change is the termination of the tie. Conversely, if the tie *x*_*ij*_ is absent, the proposed change is the creation of the tie. We denote the network resulting from the suggested change by *x*′. If the proposed change increases the value of ∑_*k*_*θ*_*k*_*s*_*k*_(*x*, *v*, *w*), the change is accepted and the new state of the network is *x*′; otherwise, it is accepted with a probability *p(x,x*′*)* that is proportional to

3 $$ {\displaystyle \begin{array}{l}p\left(x,x^{\prime}\right)\propto \exp \left\{\frac{\sum_k{\theta}_k\left[{s}_k\left(x^{\prime },v,w\right)-{s}_k\left(x,v,w\right)\right]}{T}\right\}.\\ {}\end{array}} $$

At each step, the temperature is decreased by a small factor and the algorithm stops when the value of *T* is close to 0. The last network is the most likely network (or one of the most likely networks) according to the specified ERGM. The intuition behind Eq. (3) is that the smaller the change in the linear combination of the statistics and parameters, ∑_*k*_*θ*_*k*_[*s*_*k*_(*x*′, *v*, *w*) − *s*_*k*_(*x*, *v*, *w*)], and the higher the temperature, the more likely it is for the algorithm to accept the proposed network *x*′ as the next state, even though *x*′ is a worse solution than *x*. This procedure allows the algorithm to jump to different regions of the network space and therefore to search for the optimum in the entire space. When *T* is decreased, the acceptance probability decreases; therefore, the search for the optimum concentrates in a more localised region.

For some ERGM specifications, the rationale behind choosing the most likely network does not rely merely on probabilistic theory, but it is motivated by a micro-foundation of ERGMs rested on principles of game theory (Butts 2009; Mele 2017). According to this derivation, a network is the outcome of a network formation game (Goyal 2012; Jackson 2010) in which pairs of nodes decide to create or sever ties based on a pay-off expressing the reward of the ties. The pay-off is defined as a linear combination of statistics, counting the number of configurations involving the tie considered, and parameters, representing the trade-off between the costs and benefits of a tie when it is part of the network configuration corresponding to that parameter. Under certain conditions (Monderer and Shapley 1996; Vega-Redondo 2003; Butts 2009; Mele 2017), the limiting distribution of the network formation game is the ERGM and the most likely networks are in equilibrium, that is they are networks in which none of the nodes would like to sever an existing tie or create a non-existing tie. Thus, the reconstructed networks can be thought of as attractive network configurations that would have arisen if sites had striven to form rewarding ties according to the specified ERGM.

## Example: an Application to the Pre-colonial Caribbean

To illustrate the framework we have described in the previous sections, we used data collected over a group of sites located in the north-western Greater Antilles and in the southern Lesser Antilles and we considered archaeological propositions concerning connectivity, inter-cultural contacts and exchange among those sites. The networks were generated using the simulated annealing algorithm described in the “ERGMs for Network Reconstruction” section. The initial parameters of temperature *T* was fixed at 6 and its value was decreased at each step by a factor of 0.9. The steps were repeated until *T* < 0.00001. Several runs of the algorithm were performed to check that the obtained networks were the most likely networks under the specified ERGM distribution.

**Fig. 1:**
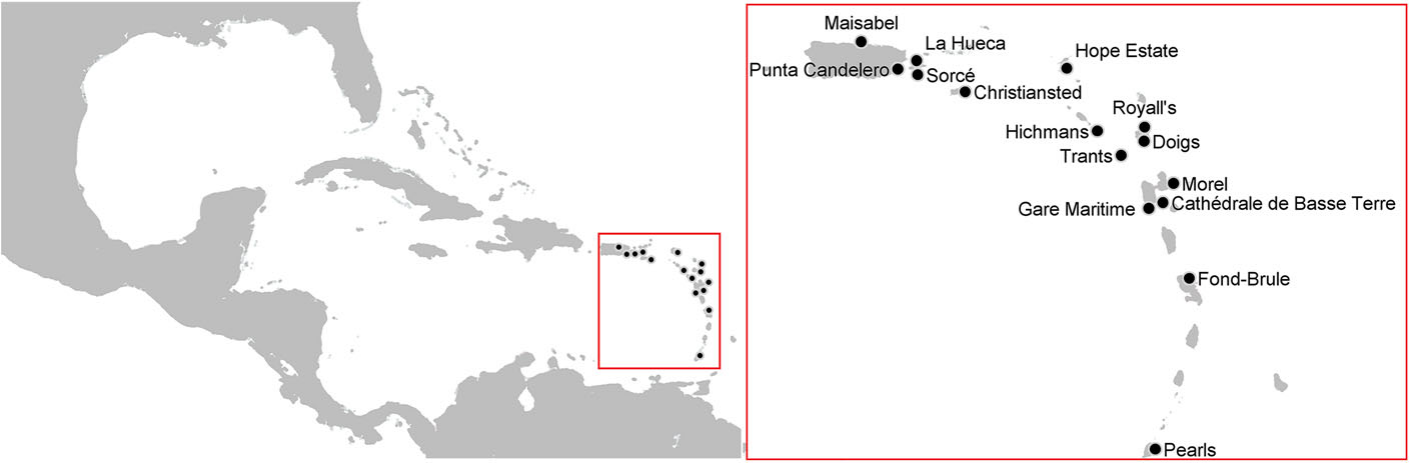
Map illustrating the geographical region and location of the sites in the data set considered. The 15 sites have been (i) suggested to be places of permanent habitation as based on their excavation reports and (ii) securely dated to the period AD 100–400 as based on a regional standard for chronometric hygiene (Fitzpatrick 2006). The red square denotes the area that appears in the subsequent figures

The considered data set is composed of a list of 15 sites that have been (i) suggested to be places of permanent habitation as based on their excavation reports and (ii) securely dated to the period AD 100–400 as based on a regional standard for chronometric hygiene (Fitzpatrick 2006). Figure 1 illustrates the geographical region and the location of the 15 sites. We refer to Hofman et al. (2014) and Hofman et al. (2019) for an in-depth description of those sites.

The period AD 100–400, hereafter referred to as the Archaic-Early Ceramic Interface (AECI) period, marks the end of a transitional process in Caribbean culture history, notably in the northern Lesser Antilles. Traditionally speaking, this marked the end of the so-called Archaic Age lifestyle, mobile hunter-gatherers living in small social units, and the advent of the Ceramic Age. However, the neolithisation process in the Caribbean, like elsewhere in the world, happened in a much more gradual way and nearly every aspect that was once argued to be introduced as part of the Ceramic Age package, from plant management to clearly articulated ceremonial life and the production of ceramics, already existed during the Archaic Age (Hofman et al. 2014, 2018, 2019). In short, the exact timing of the introduction of certain materials and practices remains hotly debated based on two models: (i) migration by which the incoming people displaced the former inhabitants and (ii) a pan-Caribbean network in which newcomers interacted with the original inhabitants and made use of existing networks. These models are furthermore based on two common traits: (i) some form of transition took place during the AECI and (ii) social networks—or their inverse (deliberate) disconnectedness—as conduits for the shift in social and cultural practices. A major touchstone in this debate is the exchange of chert and semi-precious stones raw materials, half-fabricates or finished objects (Boomert 2000; Cody 1990, 1993, Hofman et al. 2007, 2014, 2019; Keegan 2007; Rodríguez Ramos 2007).

The 15 sites selected for this study all date to the AECI. They are all characterized by ceramics of the Saladoid, Huecoid or a mix of both series (Rouse 1992) and of one or more of the named chert or semi-precious materials. The corresponding data set (see Table 1 in the supplementary material) includes information on several attributes of these sites, among them the location, the cultural affiliation and the role played in the distribution of lithic material.

The location of sites is determined by the latitude and longitude and used to compute the geographical distances among the sites. The 15 sites are located between Puerto Rico in the north-western Greater Antilles and Grenada in the southern Lesser Antilles. This area is partitioned into three sub-regions: the northern sub-region, with sites on Puerto Rico (Maisabel and Punta Candelero), Vieques (Sorcé and La Hueca), the US Virgin Island (Christiansted) and Saint Martin (Hope Estate); the central eastern sub-region with sites on Nevis (Hichmans), Antigua and Barbuda (Royall’s and Doigs), Montserrat (Trants) and Guadeloupe (Morel, Gare Maritime and Cathédrale de Basse Terre); and the southern sub-region with sites on Martinique (Fond Brule) and Grenada (Pearls). We refer to Table 1 in the supplementary material and Fig. 1 for more details.

To establish the role played by the sites in material distribution, five semi-precious stones and cherts that circulated in the considered area were taken into account: Long Island flint, amethyst, serpentinite, carnelian and Saint Martin greenstone. Conditional on the presence of a lithic material, a site was then classified as a supplier (site with lithic workshops), a supplier/intermediate, a consumer/intermediate or a consumer (site without evidence of stone working) based on the information deriving from studies of the lithic assemblage (Knippenberg 2007; Rodríguez Ramos 2007). Other information comes from excavation of the sites (see Hofman et al. (2014) and Hofman et al. (2019)). According to the quantity of finds, sites were classified into three categories: sites with a small amount (3 sites), a medium amount (6 sites) and a large amount (6 sites). Following Rouse’s classification (1992) and the composition of the ceramic assemblages, the cultural affiliation of each site has been coded into four categories: Saladoid (5 sites), Huecoid (2 sites), Saladoid and Huecoid (4 sites), Huecoid and Saladoid (4 sites).

To illustrate how ERGMs can be used to reconstruct networks between the 15 sites mentioned above, we present three different model specifications and we provide a qualitative assessment of the reconstructed networks. This assessment evaluates the coherence of the structure of the generated networks with archaeological evidence that is not directly accounted for by the model specification. In particular, we test whether certain model specifications are able to explain the presence of sites known to have functioned as hubs, *i.e.* major community gathering sites expected to be well (directly) connected to the other sites. Consequently, we consider the degree centrality of a site, *i.e.* the number of ties incident to that site, as a measure of connectedness. We expect that, in a plausible network reconstruction, the sites having higher degree must correspond to the hubs.

### Proximity Model

Many of the propositions aiming to explain connectedness underline the importance of geographical space in both formation and maintenance of network ties. Certain ways of thinking consider islands as more bounded spaces, in which connectivity between sites that are close to each other are more likely. An example of this is the “island-hopping” model (Rouse 1992) according to which movements across the Caribbean were based on a sequence of short journeys between islands. Other lines of thought consider the Caribbean Sea to have functioned as a more unbounded connector where single journeys from one destination to another took place, affording higher connectivity to all sites in the region (Keegan and Hofman 2017; Torres and Rodríguez Ramos 2008; Watters 1997). It should be noted that none of the existing theories hold extreme positions on the subject, *i.e.* either complete boundedness or complete connectivity.

Table 4 illustrates the model specification for reconstructing connectivity based on the assumption described above and on the statistics in the first and last rows of Table 2. The edge statistic counts the number of ties in the network. Large and negative values of the corresponding parameter *θ*_1_ generate sparse networks, whilst large and positive values of *θ*_1_ generate dense networks. Propositions concerning geographical proximity are modelled by the edge covariate statistic since distance between sites is a dyadic attribute. The edge covariate statistic measures the total distance spanned by all the edges presented in the network. The corresponding parameter *θ*_2_ measures the impact of distance on the existence of ties: positive values of *θ*_2_ indicate that long-distance ties are more likely to occur as the log-odds for the model in Table 4 are equal to *θ*_1_ + *θ*_2_*∙* log (*d*_*ij*_*).*

**Table 4:** ERGM specification for reconstructing connectivity. In the formulas, *x*_*ij*_ = 1 if there is a tie between *i* and *j*, and 0 otherwise; *d*_*ij*_ denotes the distance between sites *i* and *j*

Local configuration	Parameter (*θ*_*k*_)	Statistic (*s*_*k*_)
Edges	*θ*_1_	∑_*ij*_*x*_*ij*_
Edge covariate (distance)	*θ*_2_	∑_*ij*_* x*_*ij*_ log(*d*_*ij*_)

The ERGM specification in Table 4 corresponds to a spatial inhomogeneous Bernoulli random graph model (Butts 2002) which assumes that the likelihood of a tie depends on the distance according to an attenuated power law function having the form

4 $$ P\left({X}_{ij}={x}_{ij}|{D}_{ij}={d}_{ij}\right)=\frac{1}{1+{\mathrm{e}}^{-{\theta}_1-{\theta}_2\log \left({d}_{ij}\right)}}=\frac{1}{1+\alpha {d}_{ij}^{\gamma }}, $$

with $\alpha ={\mathrm{e}}^{-{\theta}_1}$ and *γ* =  − *θ*_2_. This model assumes dyadic independence and is a generalization of maximum distance networks (MDNs) (Evans et al. 2012). In MDNs, a tie is present if the distance between two sites is less than a threshold distance *D* far apart. Thus, the probability of a tie conditional on the distance is either 0 or 1 and is described by a degenerate distribution. The model in Table 4, in contrast, assigns a probability to each tie according to a decreasing function of the distance.

**Fig. 2:**
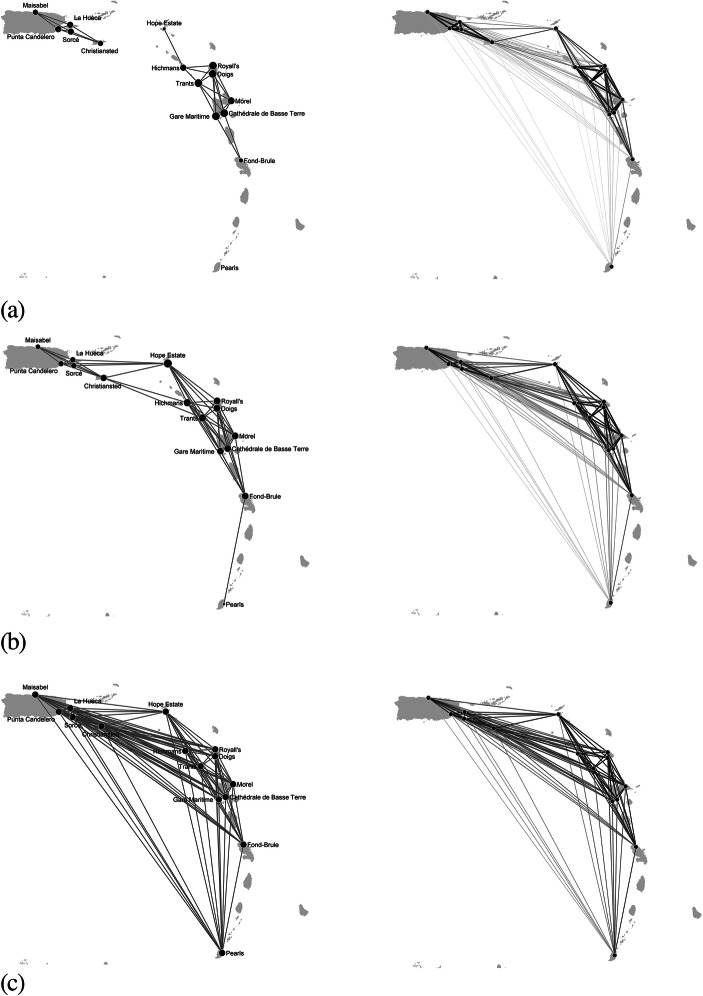
Networks reconstructed using the proximity model in Table 4. Parameter values: **(a**) *θ*_1_ = 10.98, *θ*_2_ = − 2.20; (**b)***θ*_1_ = 10.98, *θ*_2_ = − 1.91; **(c)***θ*_1_ = 10.98, *θ*_2_ = − 1.60. The size of the nodes is proportional to the node degree, *i.e.* the number of incident ties to a node. The initial value of temperature *T* was fixed at 6 and decreased by a factor of 0.9 at each step. The step of the simulated annealing algorithm was repeated until *T* < 0.00001

The left-hand side of Fig. 2 shows the most likely networks generated according to the model specification above and different values of the parameter *θ*_2_. The initial values of the parameters (*θ*_1_ = 10.98 and *θ*_2_ = − 1.91) were determined by imposing that the probability of a tie between sites 100 km (maximum daily travelling distance) far apart was equal to 0.9, whilst the probability of a tie between sites 1000 km far apart was small and equal to 0.1. The parameter *θ*_2_ was tuned to represent the archaeological propositions concerning site proximity and reachability. An increase of *θ*_2_ leads to networks characterized by the presence of long-distance ties, *θ*_1_ being equal. Therefore, when *θ*_2_ is large in absolute value and negative, the most likely networks are in line with the insularity and isolationism scenario. When *θ*_2_ is positive or small in absolute value and negative, the inter-island interaction scenario is more likely. These results are justified by the fact that high values of *θ*_2_ increase the likelihood of long-distance ties as described by Eq. (4) and visualized in the networks on the right-hand side of Fig. 2. In these networks, the colour and the size of the edges represent the probability of ties. The darker and the thicker an edge, the more likely the connectivity between two sites.

Due to the diverse structures of the reconstructed networks, Fig. 2a–c on the left-hand side differ also in the presence of hubs as suggested by differences in the size of the nodes (that are proportional to the number of incident ties) across the networks. In particular, in Fig. 2a, the hubs are the sites that belong to the largest connected component and are geographically closer to many other sites, *i.e.* Royall’s, Doigs and Trants. In Fig. 2b, when the assumption on distance is relaxed, Hope Estate is the most central site since it is geographically closer to many other sites. In Fig. 2c, none of the sites has a prevalent role with respect to the others since the resulting network is almost a complete network.

One of the hypotheses related to connectivity concerns the presence of intermediary sites leading to sequences of contacts as described by the “down the line” model (Knippenberg 2007). Generating networks coherent with this proposition requires the use of an interaction effect between two-paths and distance, so that intermediary sites act on a local scale, as shown by the model specification reported in Table 5. The local two-path statistic expresses the idea that the existence of ties between sites far apart depends on the presence of other ties connecting intermediary sites, thereby implying tie dependence. This statistic counts the number of two-paths such that connected sites are located in the same neighbourhood. For this example, we defined the neighbourhood of a site as the set of sites that are less than 200 km far apart. Large and positive values of the corresponding parameter *θ*_3_ generate networks coherent with the down the line model, whilst large and negative values of *θ*_3_ generate networks with no intermediary sites.

**Table 5:** ERGM specification for reconstructing connectivity in networks with intermediaries and cohesive sub-groups of sites. In the formulas, *d*_*ij*_ denotes the distance between sites *i* and *j*, whilst ${I}_{\left\{i,j\ \mathrm{neighbours}\right\}}$ is an indicator function, taking value 1 if *i* and *j* are in the same neighbourhood, and 0 otherwise. We defined the neighbourhood of a site as the set of sites that are less than 200 km far apart from that site

Local configuration	Parameter (*θ*_*k*_)	Statistic (*s*_*k*_)
Edges	*θ*_1_	∑_*ij*_*x*_*ij*_
Edge covariate (distance)	*θ*_2_	∑_*ij*_*x*_*ij*_ log(*d*_*ij*_)
Local two-path	*θ*_3_	${\sum}_{ij h}{x}_{ij}\backslash \mathrm{mathbbm}{I}_{\left\{i,j\ neighbors\right\}}{x}_{jh}\backslash \mathrm{mathbbm}{I}_{\left\{j,h\ neighbors\right\}}$

**Fig. 3:**
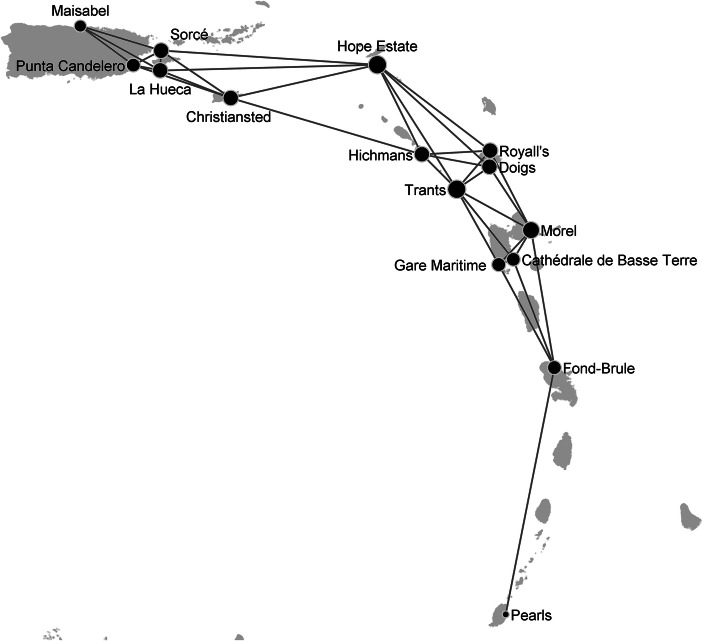
Network reconstructed using the model in Table 5. Parameter values: *θ*_1_ = 9.0, *θ*_2_ = 0.5, *θ*_3_ = − 1.6. The size of the nodes is proportional to the node degree, *i.e.* the number of incident ties to a node. The initial value of temperature *T* was fixed at 6 and decreased by a factor of 0.9 at each step. The step of the simulated annealing algorithm was repeated until *T* < 0.00001

An example of network coherent with the down the line model is shown in Fig. 3. Compared to the network in Fig. 2b, the presence of intermediary sites bridging the connections between far-away sites reduces the number of long-distance ties. For instance, there are no more direct ties from Hope Estate and Gare Maritime to Morel and Cathédrale de Basse Terre due to the presence of the intermediary sites Trants, Royall’s and Doigs. In this network, the most central sites (*e.g.* Hope Estate, Trants and Morel) are located in the central eastern sub-region as denoted by the size of the corresponding nodes.

**Fig. 4:**
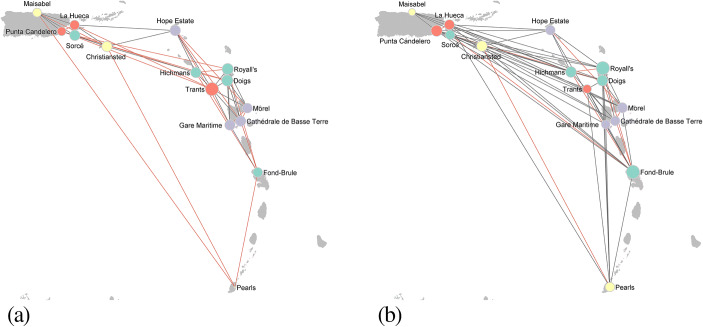
Network reconstructed using the inter-cultural model in Table 3. Parameter values: **(a**) *θ*_1_ = 2, *θ*_2_ = − 0.3, *θ*_3_ = − 0.3, *θ*_4_ = − 0.6, *θ*_5_ = − 0.9, *θ*_6_ = − 0.3, *θ*_7_ = − 0.6, *θ*_8_ = − 0.6; **(b**) *θ*_1_ = 2.0, *θ*_2_ = − 0.3, *θ*_3_ = 0.1, *θ*_4_ = 0.2, *θ*_5_ = 0.3, *θ*_6_ = 0.1, *θ*_7_ = 0.2, *θ*_8_ = 0.2. The colour of the nodes represents their cultural affiliation: Saladoid (green), Saladoid and Huecoid (yellow), Huecoid and Saladoid (violet), Huecoid (pink). The colour of the ties indicates whether a tie exists between two sites having the same cultural affiliation (red) or having a different cultural affiliation (grey). The size of nodes is proportional to the node degree, *i.e.* the number of incident ties to a node. The initial value of temperature *T* was fixed at 6 and decreased by a factor of 0.9 at each step. The step of the simulated annealing algorithm was repeated until *T* < 0.00001

**Fig. 5:**
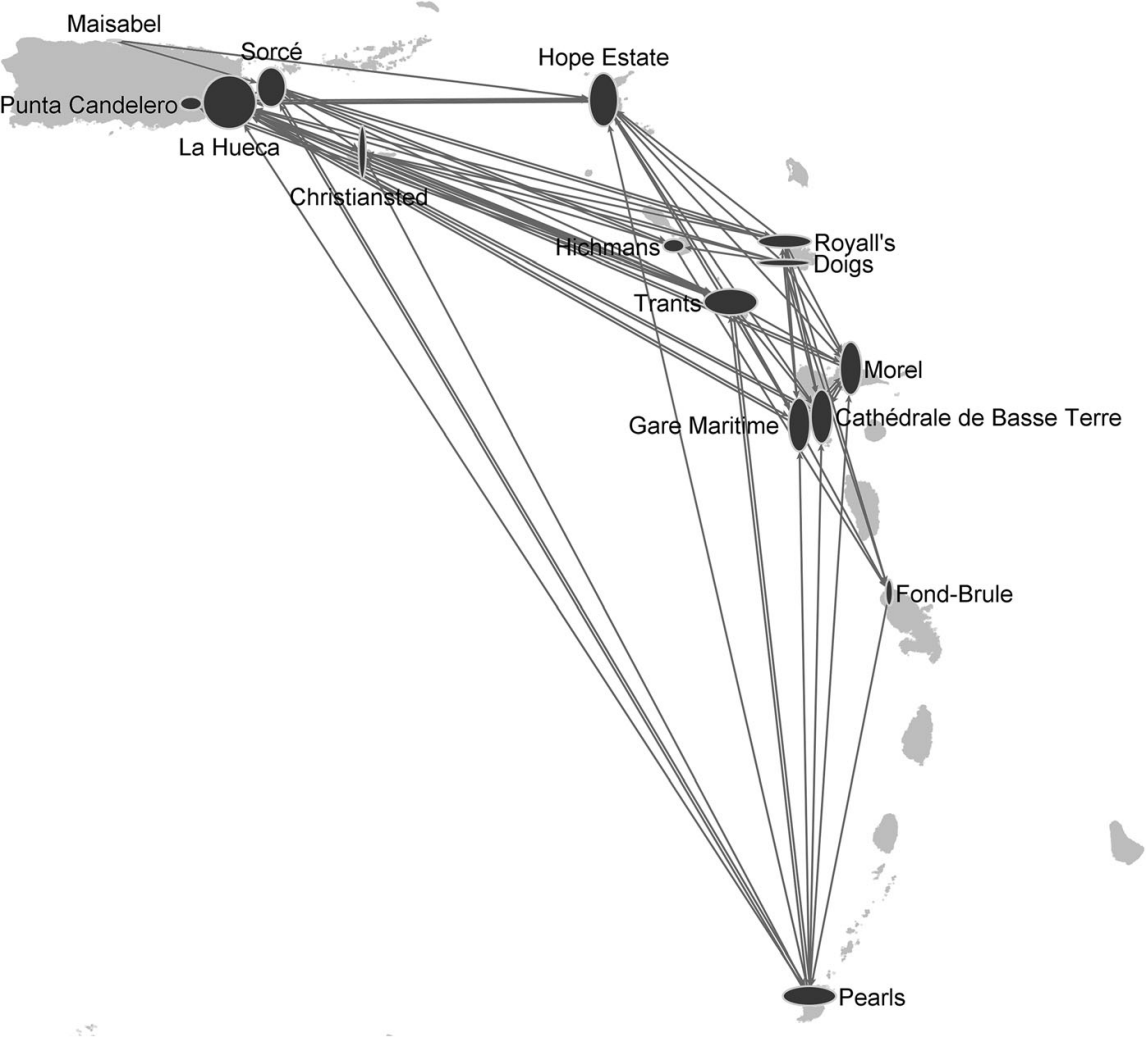
Exchange network reconstructed using the model in Table 7. Parameter values: *θ*_1_ = − 4.0, *θ*_2_ = − 0.1, *θ*_3_ = 1.0, *θ*_4_ = 1.0, *θ*_5_ = 0.6, *θ*_6_ = 0.6, *θ*_7_ = 0.1, *θ*_8_ = 0.1, *θ*_9_ = *θ*_10_ = *θ*_11_ = *θ*_12_ = *θ*_13_ = 1, *θ*_14_ = − 1.5, *θ*_15_ = − 3, *θ*_16_ = − 4.5, *θ*_17_ = − 1.5, *θ*_18_ = − 3, *θ*_19_ = − 1.5. The width and the height of a node are proportional to its out-degree (*i.e.* the number of outgoing ties), and in-degree (*i.e.* the number incoming ties), respectively. The initial value of temperature *T* was fixed at 6 and decreased by a factor of 0.9 at each step. The step of the simulated annealing algorithm was repeated until *T* < 0.00001

### Proximity and Inter-cultural Model

In Caribbean Archaeology, the type of theories that argue for lower or no cohesion between sites focusses on the role of cultural boundaries constricting contacts in AECI networks. Different pottery decorations and ceramic assemblages are used as evidence for the presence of different groups of people and cultures, who competed with and culturally supplanted each other. Long-range network contacts would only exist with groups with the same culture or, in Rouse’s (1992) terminology, within the same “people”.

Several archaeologists explained the presence of bounded groups with different migration waves into the Caribbean islands (see, for instance, Veloz Maggiolo (1991) and Zucchi (1990)). At the core of this is the idea that one or more peoples migrated into the northern Lesser Antilles in the first millennium BC and their movement was limited by the social distance represented by diverse cultural affiliations. This social distance led to the formation of strictly bounded social groups, as reflected in ceramic assemblages, with their own regional and inter-regional contact networks. These only gradually converged in the centuries after the migration had taken place.

In opposition to these older ideas are several theories that consider the AECI to be a period of networks operating at a pan-Caribbean scale with a high degree of inter-regional mobility and multi-cultural communities, despite differences in culture affiliation (*e.g.* Hofman et al. 2010, 2011; Rodríguez Ramos et al. 2010). Whilst these theories are probably better suited to understand an archaeological record that showcases a unity in diversity (Mol 2014), many are vested in archaeologically unobservable or unspecified effects, such as family ties or the unspecified concept of “network” (Hardy 2008; Keegan 2004; Keegan and Hofman 2017).

None of the previous models for network reconstruction account for presence or absence of cultural boundaries, and therefore cannot be used in this context. Conversely, ERGMs provide a set of node covariate statistics to model a more general notion of (dis) similarity in cultural affiliation. Those statistics are added to those present in Table 4 to control for geographically proximity. Thus, this example demonstrates that ERGMs can account for more propositions at the same time.

Let *v* be the variable describing the cultural affiliation of the sites. This variable takes four categories: Saladoid, Huecoid, Saladoid and Huecoid, Huecoid and Saladoid. Table 6 reports one possible model specification to represent networks coherent with the assumptions on cultural homophily and distance. Using the heuristic interpretation of ERGMs, the corresponding parameters *θ*_3_*, …, θ*_8_ can be interpreted as the change in the log-odds of a tie being present between sites having a different cultural affiliation and sites having the same cultural affiliation. Thus, a model based on theory of cultural boundaries is characterized by negative values of those parameters, whilst a model coherent with the theory of inter-cultural contacts is characterized by positive values of those parameters. In fact, positive values of the parameters *θ*_3_, …, *θ*_8_ lead to a positive contribution to the log-odds in Eq. (2), thereby suggesting that ties between sites having different cultural affiliation are more likely to be present than absent.

**Table 6:** An example of ERGM specification for reconstructing inter-cultural networks. The node attribute *v* represents the cultural affiliation. In the formulas, *d*_*ij*_ denotes the distance between sites *i* and *j*, whilst $\backslash \mathrm{mathbbm}{I}_{\left\{{v}_i=\mathrm{a},{v}_j=\mathrm{b}\right\}}$ is an indicator function, taking value 1 if the cultural affiliation *v*_*i*_ of site *i* is *a* and the cultural affiliation *v*_*j*_ of site *j* is *b*

Local configuration	Parameter (*θ*_*k*_)	Statistic (*s*_*k*_)
Edges	*θ*_1_	∑_*ij*_*x*_*ij*_
Edge covariate (distance)	*θ*_2_	∑_*ij*_*x*_*ij*_ log(*d*_*ij*_)
Homophily (cultural similarity)	*θ*_3_	${\sum}_{ij}{x}_{ij}\backslash \mathrm{mathbbm}{I}_{\left\{{v}_i=\mathrm{Saladoid},{v}_j=\mathrm{Saladoid}\ \mathrm{and}\ \mathrm{Huecoid}\right\}}$
	*θ*_4_	${\sum}_{ij}{x}_{ij}\backslash \mathrm{mathbbm}{I}_{\left\{{v}_i=\mathrm{Saladoid},{v}_j=\mathrm{Huecoid}\ \mathrm{and}\ \mathrm{Saladoid}\right\}}$
	*θ*_5_	${\sum}_{ij}{x}_{ij}\backslash \mathrm{mathbbm}{I}_{\left\{{v}_i=\mathrm{Saladoid},{v}_j=\mathrm{Huecoid}\right\}}$
	*θ*_6_	${\sum}_{ij}{x}_{ij}\backslash \mathrm{mathbbm}{I}_{\left\{{v}_i=\mathrm{Saladoid}\ \mathrm{and}\ \mathrm{Huecoid},{v}_j=\mathrm{Huecoid}\ \mathrm{and}\ \mathrm{Saladoid}\right\}}$
	*θ*_7_	${\sum}_{ij}{x}_{ij}\backslash \mathrm{mathbbm}{I}_{\left\{{v}_i=\mathrm{Saladoid}\ \mathrm{and}\ \mathrm{Huecoid},{v}_j=\mathrm{Huecoid}\right\}}$
	*θ*_8_	${\sum}_{ij}{x}_{ij}\backslash \mathrm{mathbbm}{I}_{\left\{{v}_i=\mathrm{Huecoid}\ \mathrm{and}\ \mathrm{Saladoid},{v}_j=\mathrm{Huecoid}\right\}}$

Reconstructed networks for different values of the parameters *θ*_3_, …, *θ*_8_ are shown in Fig. 4. Both networks are characterized by the presence of short-distance ties due to the negative values of the distance parameter. However, whilst Fig. 4a provides a picture of a network coherent with Rouse’s (1992) hypothesis of the presence of cultural boundaries, Fig. 4b shows a plausible network characterized by absence of cultural boundaries. The proportion of ties between sites having a different cultural affiliation (grey ties in Fig. 4) is indeed 0.41 for the network in Fig. 4a, and 0.77 for the network in Fig. 4b.

The two networks differ also in their structures. The network in Fig. 4b is denser and characterized by the presence of long-distance ties. In Fig. 4a, Trants, Royall’s and Doigs are the most central sites since they are located close to other sites having a similar cultural affiliation. Conversely, Fund-Brule, Royall's and Punta Candelero are the most important sites in Fig. 4b obtained by assuming cultural affiliation heterophily.

### Proximity, Inter-cultural and Exchange Model

We consider now the reconstruction of exchange networks to provide an example of ERGM specification for directed relations and introduce effects that have not been considered so far. The distribution of semi-precious stones and cherts that circulated in the considered area is the outcome of exchange relations from one site, referred to as the “sender”, to another site, referred to as the “receiver”. Therefore, exchange relations are characterized by directionality.

A simple model for reconstructing exchange is specified by assuming that exchange ties depend on the proximity between sites (Fitzpatrick 2004), similarity in material culture and the role played by the sites in the distribution of the lithic material. In particular, Hofman et al. (2014) suggested that supplier sites (*i.e.* sites with lithic workshops) are more likely to be senders, whilst consumer sites (*i.e.* sites without evidence of stone working) are more likely to be receivers.

These assumptions are included in the model by considering the covariate activity and covariate popularity statistics as shown in Table 7. Let *u* and *z* be the variables describing the number of lithic sources of which a site is a supplier and a consumer, respectively. The covariate activity and covariate popularity statistics measure the number of outgoing ties for supplier sites and the number of incoming ties for consumer sites, respectively. Thus, positive values of *θ*_3_ indicate that supplier sites tend to have more outgoing ties than consumer sites, and positive values of *θ*_4_ indicate that consumer sites tend to have more incoming ties than supplier sites.

**Table 7:** An example of ERGM specification for reconstructing exchange networks based on distance (*d*_*ij*_), material culture (*v*), the role of sites in the redistribution of lithic material (*z* for supplier, *u* for consumer and *w* for intermediate), the quantity of finds in a site (*q*) and the directionality of the exchange from supplier to consumer sites (*s*_*ij*_)

Local configuration	Parameter (*θ*_*k*_)	Statistic (*s*_*k*_)
Edges	*θ*_1_	∑_*ij*_*x*_*ij*_
Edge covariate (distance)	*θ*_2_	∑_*ij*_*x*_*ij*_ log(*d*_*ij*_)
Covariate activity	*θ*_3_	∑_*ij*_*x*_*ij*_*z*_*i*_
Covariate popularity	*θ*_4_	∑_*ij*_*x*_*ji*_*u*_*i*_
Covariate activity	*θ*_5_	∑_*ij*_*x*_*ij*_*w*_*i*_
Covariate popularity	*θ*_6_	∑_*ij*_*x*_*ji*_*w*_*i*_
Covariate activity	*θ*_7_	∑_*ij*_*x*_*ij*_*q*_*i*_
Covariate popularity	*θ*_8_	∑_*ij*_*x*_*ij*_*q*_*j*_
Edge covariate (source)	*θ*_9_, …, *θ*_13_	∑_*ij*_*x*_*ij*_*s*_*ij*_
Homophily (cultural similarity)	*θ*_14_	${\sum}_{ij}{x}_{ij}\backslash \mathrm{mathbbm}{I}_{\left\{{v}_i=\mathrm{Saladoid},{v}_j=\mathrm{Saladoid}\ \mathrm{and}\ \mathrm{Huecoid}\right\}}$
	*θ*_15_	${\sum}_{ij}{x}_{ij}\backslash \mathrm{mathbbm}{I}_{\left\{{v}_i=\mathrm{Saladoid},{v}_j=\mathrm{Huecoid}\ \mathrm{and}\ \mathrm{Saladoid}\right\}}$
	*θ*_16_	${\sum}_{ij}{x}_{ij}\backslash \mathrm{mathbbm}{I}_{\left\{{v}_i=\mathrm{Saladoid},{v}_j=\mathrm{Huecoid}\right\}}$
	*θ*_17_	${\sum}_{ij}{x}_{ij}\backslash \mathrm{mathbbm}{I}_{\left\{{v}_i=\mathrm{Saladoid}\ \mathrm{and}\ \mathrm{Huecoid},{v}_j=\mathrm{Huecoid}\ \mathrm{and}\ \mathrm{Saladoid}\right\}}$
	*θ*_18_	${\sum}_{ij}{x}_{ij}\backslash \mathrm{mathbbm}{I}_{\left\{{v}_i=\mathrm{Saladoid}\ \mathrm{and}\ \mathrm{Huecoid},{v}_j=\mathrm{Huecoid}\right\}}$
	*θ*_19_	${\sum}_{ij}{x}_{ij}\backslash \mathrm{mathbbm}{I}_{\left\{{v}_i=\mathrm{Huecoid}\ \mathrm{and}\ \mathrm{Saladoid},{v}_j=\mathrm{Huecoid}\right\}}$

To account for the presence of intermediary sites and the assumption that those sites might have both outgoing and incoming ties, we defined a variable *w* describing the number of lithic sources of which a site is an intermediary. We then included the corresponding covariate activity and covariate popularity statistics in the model in Table 7. Following the same reasoning, we added to the model the covariate activity and covariate popularity statistics for the variable *q* describing the quantity of finds in a site. The corresponding parameters *θ*_5_ and *θ*_7_ are interpreted as the parameter *θ*_3_, whilst the parameters *θ*_6_ and *θ*_8_ are interpreted as the parameter *θ*_4_*.*

Finally, to account for the exchange flow, we assumed that ties between suppliers and consumers of the same lithic material were more likely than ties between sites being both suppliers or both consumers of the same lithic material. This assumption was incorporated in the model by using an edge covariate statistic for each lithic material. The edge covariate *s*_*ij*_ for a specific source takes value 1 if site *i* is a supplier and site *j* is a consumer, and 0 otherwise. This definition of the edge covariate allows to account for the directionality of the exchange from supplier to consumer sites. Positive values of parameters *θ*_9_, …, *θ*_13_ suggest that ties from supplier to consumer sites of the same lithic material were more likely.

The most likely network coherent with the propositions of Hofman et al. (2014) is depicted in Fig. 5. The network is quite dense and characterized by long-distance ties suggesting that the Caribbean Sea has functioned as an unbounded connector where single journeys from one destination to another took place, affording higher connectivity to all sites in the region. The size of the node indicate the role played by the sites in the exchange network. Mainly supplier sites (*i.e.* sites that are suppliers of multiple lithic materials, such as Trants and Royall’s suppliers of Long Island flint and Carnelian) have more outgoing ties than incoming ties and therefore are represented by nodes with width greater than height. On the contrary, mainly consumer sites (*i.e.* sites that are consumers of multiple lithic materials, such as Hope Estate—consumer of Long Island flint, amethyst, serpentinite and carnelian—and Morel—consumer of Long Island flint, amethyst, carnelian and Saint Martin greenstone) have more incoming ties than outgoing ties and therefore are represented by nodes with height greater than width. Rounded nodes denote sites that are both suppliers and consumers without any prevalence. The computation of the degree of the nodes (*i.e.* the sum of the in-degree and out-degree) suggests that the network is characterized by three main hubs La Hueca, Trants and Pearls which play the role of the three major community gathering sites in the northern, central-eastern and southern sub-regions.

A more complex model can be specified to account for hierarchy among the sites. As discussed in the “Statistics” section, networks coherent with this assumption can be obtained by adding the transitive triads and the 3-cycles statistics to the model specified in Table 7.

## Conclusion

The archaeological literature provides a large variety of assumptions concerning interaction mechanisms between archaeological contexts. Those assumptions have been used to infer the structure of past networks by specifying models that reflect the available archaeological knowledge.

In this paper, we considered tie-based models, specifically exponential random graph models (ERGMs) which offer a general framework that may be applied to infer the structure of ancient networks in diverse archaeological settings. Compared to previous models, the formulation of ERGMs does not hinge on the specific assumptions, the time period, the geographical area or the type of relation considered. Moreover, ERGMs enable the reconstruction of networks based on a large variety of propositions, ranging from assumptions based on dyadic independence to those assuming tie dependence and accounting for node or dyadic attributes. Thus, the application of ERGMs opens up the investigation of scenarios that cannot be explored by previous models.

The application of ERGMs to reconstruct archaeological networks is a probabilistic approach and contrasts with the deterministic approach of maximum distance networks, proximal point analysis, and gravity models. In those models, the presence of a relationship between two sites is defined by a rule stating which ties exist; therefore, the outcome of those models is fixed. The probabilistic approach of ariadne and ERGMs, in contrast, has the advantage of assigning probabilities to the generate networks and thus to partially control for the incomplete information derived from the data.

The probabilistic approach of ERGMs requires two fundamental steps: (i) the specification of the model and (ii) the generation of plausible networks.

The model specification consists of choosing the statistics and the parameter values. The selection of the statistics is based on the archaeological assumptions and their encoding into local network configurations, *i.e.* graphical representations of nodes and ties. In Table 2 and Table 3, we provided a summary of the most common network configurations along with the corresponding ERGM statistics, and related them to some of the archaeological hypotheses that have been formulated when analysing relations among different archaeological contexts. The choice of the parameter values was based on both archaeological knowledge and the tuning procedure described in the “ERGMs for Network Reconstruction” section. Whilst the archaeological assumptions provide information on the sign of the parameter values (positive if there is a tendency for the tie mechanism implied by the assumption, but negative otherwise), the tuning avoids the generation of uninformative network structures, such as empty and full networks.

Given a fully specified ERGM, possible network scenarios are generated by maximising the corresponding probability distribution. Due to the high number of networks that can be defined over a set of nodes, the maximisation problem is difficult and thus an optimisation algorithm based on simulated annealing is used. The choice of considering the most likely network as a plausible reconstruction is justified by statistical principles and the derivation of the ERGM distribution from concepts of game theory. For some specifications, ERGMs are the limiting distribution of a process in which nodes form beneficial ties as quantified by a pay-off function measuring the trade-off between the costs and benefits of ties. The maxima of this limiting distribution are desirable networks, *i.e.* configurations where none of the nodes would form a non-existing tie or sever an existing tie. Thus, the reconstructed networks can be thought of as attractive network configurations that would have arisen if sites had striven to form beneficial ties according to the specified ERGM.

Although, ERGMs provide a flexible method that can be applied to many archaeological contexts, the illustrated framework has some limitations.

Firstly, the inferred networks provide only one picture of the network in the past. Even if the generated networks can be interpreted as desirable network configurations emerging from a process in which sites form and sever ties according to their costs and benefits, the outcome of the framework is essentially static. Therefore, this approach cannot be used to investigate network evolution or the diffusion of practices and innovations. For this purpose, agent-based, network diffusion and dynamic network models are more suitable than ERGMs. Moreover, due to the difficulty of the optimisation problem, the networks generated are binary networks, indicating only if a tie was present or absent, and they do not provide any information on the strength of the ties among the sites.

Secondly, the procedure illustrated allows to compute the probability of a tie only in some trivial cases. For instance, in the illustrative example, we demonstrated that the likelihood of a tie can be computed using an attenuated power law function when connectivity is determined only by distance. However, when the specification of the ERGM includes triadic effects, such as the model accounting for intermediary sites, then the best we can do is compute tie probabilities conditional on the reconstructed network. We cannot provide the unconditional probability of a tie as we can in models for tie independence.

Finally, as with all the other models for network reconstruction, the networks resulting from ERGMs are sensitive to missing data. The structure of a reconstructed network might indeed vary when new sites are considered. The use of Bayesian procedures might offer a better approach to dealing with the incompleteness of the archaeological data. Bayesian statistics have been already used to model partially observed networks (Handcock and Gile 2010; Koskinen et al. 2010), to impute missing data in network studies (C. Wang et al. 2016), and to infer links given noisy or proxy data (Newman 2018b, 2018c). Due to the high level of uncertainty and incompleteness of the archaeological data, those approaches, albeit promising, need to be further developed to be used to reconstruct past networks.

We illustrated the applicability of the framework by reconstructing networks between 15 sites located between Puerto Rico, in the north-western Greater Antilles, and Grenada, in the southern Lesser Antilles, during the period AD 100–400, here referred to as the Archaic-Early Ceramic Interface period (AECI period). In particular, we considered several relations and some model specifications to demonstrate the operation of the framework and the flexibility of the model in a period and cultural context of probable inter-cultural contact. The networks generated indicate that the assumptions about tie formation and, consequently, the specified model largely influence the structure of the reconstructed network; thus, these networks provide a variety of different scenarios that were qualitatively assessed by evaluating the coherence of the structure of the generated networks with archaeological evidence that was not directly accounted for by the model specification. In this paper, we tested whether the considered model specifications were able to explain the presence of sites known to have functioned as hubs but other criteria might be used according to the available archaeological information.

All the networks generated for this study provide credence to a balanced view of Caribbean inter-community interactions in the AECI. However, the networks generated using only propositions related to distance and cultural homophily are not plausible network reconstructions since they do not reflect the archaeological evidence on the importance of some sites in the past. Specifically, none of these networks points to the existence of major community gathering sites located in the northern, central-eastern and southern sub-regions of the area considered. Adding information on the distribution of lithic materials and the quantity of finds provides a more faithful reconstruction of the network of contacts between the considered sites. In fact, the resulting network underlines previous ideas on tight local lithic networks combined with a moderate amount of connectivity at the level of the region and supports the archaeological evidence that La Hueca, Trants and Pearls were the three major gathering sites during the AECI. This finding indicates that the structure of networks in the AECI period was determined by multiple interdependent mechanisms which go beyond hypotheses about distance and cultural homophily. Moreover, the coupling of archaeological provenance data, such as the AECI distribution of local lithic raw materials, with specific archaeological theories of network effects is profitable in our illustrative example and easy to implement within the ERGM framework. Regardless, we should keep in mind that this is only one data source; therefore, the results of the model may not be robust when confronted with expanded and new provenance data.

The illustrative example also indicates where the value of ERGMs for archaeological network reconstruction resides. Regardless of region, time period or data set quality and quantity, ERGMs require a formal exploration of theories of network foundation and development. This requirement goes against the grain of “informal archaeological network studies” in the Caribbean and elsewhere, where cultural histories have been built on at best tacitly understood links between material culture and social networks or at worst fuzzy ones. The ERGM framework (1) necessitates the formation of theories based on well-understood and clearly communicable network effects, such as geographic proximity, cultural homophily or the(re-)distribution of raw materials, and (2) allows for the exploration of these effects, as well as their interdependency, which can then be used to scaffold data-driven theories. In short, this study advocates for the adoption or creation of more formalised network theories, as well as data in archaeology at large, and underscores their value. Doing so in the case of the Caribbean may provide new and more specific insights into connectivity in the AECI and other periods.

## Electronic Supplementary Material

ESM 1 (PDF 135 kb)
