# Supplementary material for: A Framework for Reconstructing Archaeological Networks Using Exponential Random Graph Models
Source: J Archaeol Method Theory. 2019 Aug 19;27(2):192–219. doi: 10.1007/s10816-019-09423-z (PMC7252583; doi:10.1007/s10816-019-09423-z)
Supplement: Supplementary file 1 — (PDF 135 kb) [file 10816_2019_9423_MOESM1_ESM.pdf]

Supplementary material

Site	Sub-region	Island	Longitude	Latitude	Long Island	Serpentinite	St.M.Greenstone	Carnelian	Amethyst	Quantity	Cultural Affiliation
Maisabel	North	Puerto Rico	-66.38	18.49	None	Supplier	Consumer	None	None	Medium	Saladoid and Huecoid
Punta Candelero	North	Puerto Rico	-65.79	18.09	Consumer	Supplier	Consumer	None	None	Medium	Huecoid
La Hueca	North	Vieques	-65.48	18.12	Consumer/ Intermediate	Supplier/ Intermediate	Consumer/ Intermediate	Consumer	Consumer	Small	Huecoid
Sorcé	North	Vieques	-65.48	18.12	Consumer	Supplier/ Intermediate	Consumer/ Intermediate	Consumer	Consumer	Small	Saladoid
Christiansted	North	US Virgin Island	-64.7	17.75	Consumer	None	Consumer	None	Consumer	Large	Saladoid and Huecoid
Hope Estate	North	Saint Martin	-63.05	18.09	Consumer	Consumer	Supplier	Consumer	Consumer	Medium	Huecoid and saladoid
Hichmans	Central-east	Nevis	-62.55	17.13	Supplier/ Intermediate	None	Consumer	None	None	Large	Saladoid
Doigs	Central-east	Antigua and Barbuda	-61.79	17.02	Supplier/ Intermediate	None	Consumer	Supplier	Not specified	Medium	Saladoid
Royall's	Central-east	Antigua and Barbuda	-61.79	17.16	Supplier/ Intermediate	Consumer	Consumer	Supplier	None	Medium	Saladoid
Trants	Central-east	Montserrat	-62.16	16.77	Supplier/ Intermediate	Consumer	Consumer	Supplier	Consumer	Medium	Saladoid and Huecoid
Gare Maritime	Central-east	Guadeloupe	-61.64	15.97	Consumer	None	Consumer	Consumer	Consumer	Large	Huecoid and saladoid
Cathédrale de Basse Terre	Central-east	Guadeloupe	-61.57	15.99	Consumer	None	Consumer	Consumer	Consumer	Large	Huecoid and saladoid
Morel	Central-east	Guadeloupe	-61.33	16.33	Consumer	None	Consumer	Consumer	Consumer	Large	Huecoid and saladoid
Fond-Brule	South	Martinique	-61.07	14.84	Consumer	None	Consumer	None	None	Large	Saladoid
Pearls	South	Grenada	-61.61	12.15	None	Consumer	Consumer	None	Supplier	Small	Saladoid and Huecoid

Table 1 - *Data set used for the illustrative case study. The data set contains information on 15 sites located between Puerto Rico in the northwestern Greater Antilles and Grenada in the southern Lesser Antilles. The first two columns report information on the geographical area of the sites: the Antilles sub-regions (Sub-region) and the islands (Island) on which the sits are located. The next two columns provide the exact location of the sites in terms of latitude (Latitude) and longitude (Longitude). The next five columns contain information on the role played by the sites in the distribution of five lithic materials: Long Island flint (Long Island), serpentinite (Serpentinite), Saint Martin greenstone (St. Martine Greenstone), carnelian (Carnelian), and amethyst (Amethyst). The classification of the sites into supplier (site with lithic workshops), supplier/intermediate, consumer/intermediate, and consumer (site without evidence of stone working) is based on studies of the lithic assemblages of the sites* (*Knippenberg 2007; Rodríguez Ramos 2007). The last two columns of the data set report information on the quantity of finds (Quantity) and the composition of the ceramic assemblages (Cultural affiliation). Those information comes from excavation (see Hofman et al.* (2014) *and Hofman et al. (2019)). The data set was created by Corinne Hofman and Angus Mol.*
